# Synthesis, structural studies and Hirshfeld surface analysis of 2-[(4-phenyl-1*H*-1,2,3-triazol-1-yl)methyl]pyridin-1-ium hexa­kis­(nitrato-κ^2^*O*,*O*′)thorate(IV)

**DOI:** 10.1107/S2056989024006352

**Published:** 2024-07-05

**Authors:** Shalini Rangarajan, Sonu Sheokand, Victoria L. Blair, Glen B. Deacon, Maravanji S. Balakrishna

**Affiliations:** a IITB-Monash Research Academy Indian Institute of Technology Bombay,Mumbai 400076 India; b Phosphorus Laboratory Department of Chemistry Indian Institute of Technology, Bombay,Mumbai 400076 India; c School of Chemistry, Monash University, Melbourne Clayton, Victoria 3800, Australia; Universidad de Los Andes, Venezuela

**Keywords:** crystal structure, thorium complex, triazole framework, Hirshfeld surface analysis

## Abstract

Reaction of thorium(IV) nitrate with 2-[(4-phenyl-1*H*-1,2,3-triazol-1-yl)meth­yl]pyridine (*L*) yielded (*L*H)_2_[Th(NO_3_)_6_] or (C_14_H_13_N_4_)_2_[Th(NO_3_)_6_] (**1**), instead of the expected mixed-ligand complex [Th(NO_3_)_4_*L*_2_], which was detected in the mass spectrum of **1**. In the structure, the [Th(NO_3_)_6_]^2−^ anions display an icosa­hedral coordination geometry and are connected by *L*H^+^ cations through C—H⋯O hydrogen bonds. The *L*H^+^ cations inter­act *via* N—H⋯N hydrogen bonds. Hirshfeld surface analysis indicates that the most important inter­actions are O⋯H/H⋯O hydrogen-bonding inter­actions, which represent a 55.2% contribution.

## Abstract 2

The complex 2-[(4-phenyl-1*H*-1,2,3-triazol-1-yl)meth­yl]pyridin-1-ium hexa­kis­(nitrato-*O*,*O*′)thorate was synthesized from layered solutions of Th(NO_3_)_4_·5H_2_O and 2-[(4-phenyl-1*H*-1,2,3-triazol-1-yl)meth­yl]pyridine (*L*).

## 1. Chemical context

The nitrate ion with its small chelate or bite angle has a low steric footprint and is able to stabilize high coordination numbers. Thus 12-coordinate [Th(NO_3_)_6_]^2−^ has been isolated and structurally characterized with a variety of counter-cations such as: phen_2_H^+^ (phen = 1,10-phenanthroline; Amani & Tayebee, 2013 ▸), bpyH_2_^2+^ (bpy = 4,4′-bi­pyridine; Rammo *et al.*, 1994 ▸) and NH_4_^+^ (Spirlet *et al.*, 1992 ▸), acetyl­pyridinium(thio­semicarbazone) (Abram *et al.*, 1999 ▸), 2,2′-bipyridinium (Kumar & Tuck, 1984 ▸), [5,10,15,20-tetra­kis­(pyridinium-4-yl)porphyrin] (Mishra *et al.*, 2019 ▸), 1-ethyl-3-methyl-1*H*-imidazol-3-ium (Kelley *et al.*, 2020 ▸), among others (see *Database survey* section). Other inter­esting complexes are bis­(oxonium di­cyclo­hexano-18-crown-6) hexa­kis­(nitrato-*O*,*O*′)-thorium(IV) where the counter-cation is H_3_O^+^ (Wang *et al.*, 1988 ▸) and bis­[trinitrato-tetra­kis­(tri­methyl­phosphine oxide)thorium(IV)] hexa­nitratothorium(IV) (Alcock *et al.*, 1978 ▸) where both the anion and the cation are Th^IV^ complex ions.

Hexanitratothorate [Th(NO_3_)_6_]^2−^ and its analogous species are important in the speciation and separation of actinoid complexes in nitric acid (Zhang *et al.*, 2017 ▸; Surbella *et al.*, 2018 ▸; Takao *et al.*, 2019 ▸, 2020 ▸; Reilly *et al.*, 2012 ▸; Matonic *et al.*, 2002 ▸; Crawford *et al.*, 2009 ▸; Rebizant *et al.*, 1988 ▸).
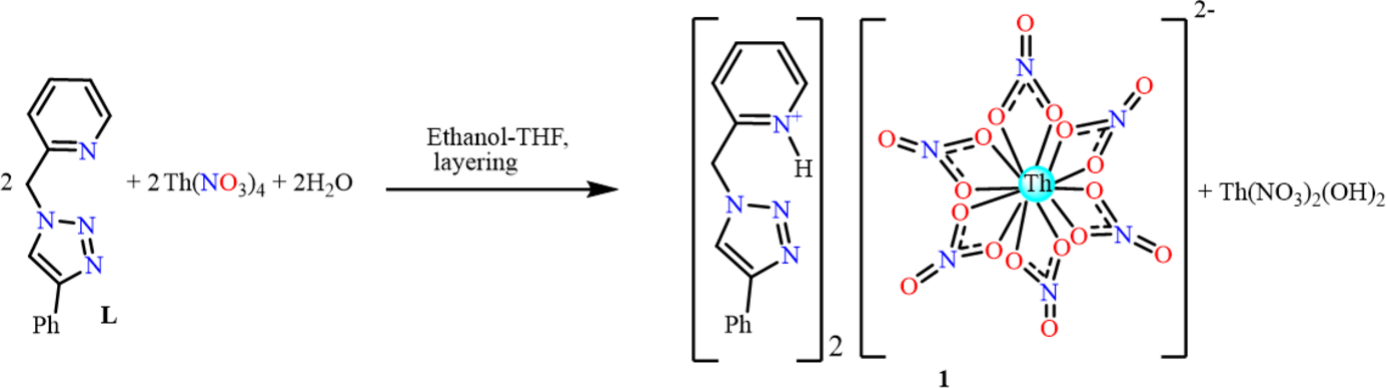


Reaction of Th(NO_3_)_4_·5H_2_O with 2-[(4-phenyl-1*H*-1,2,3-triazol-1-yl)meth­yl]pyridine (*L*) resulted in the formation of the nitrato complex (*L*H)_2_[Th(NO_3_)_6_] (**1**), instead of [Th(NO_3_)_4_*L*_2_], analogous to complexes of functionalized chelating pyridine-based ligands (Gephart *et al.*, 2009 ▸; Xiao *et al.*, 2014 ▸). The structure of complex **1** was established by X-ray crystallography, IR and mass spectroscopic data.

## 2. Structural commentary

**Figure 1:**
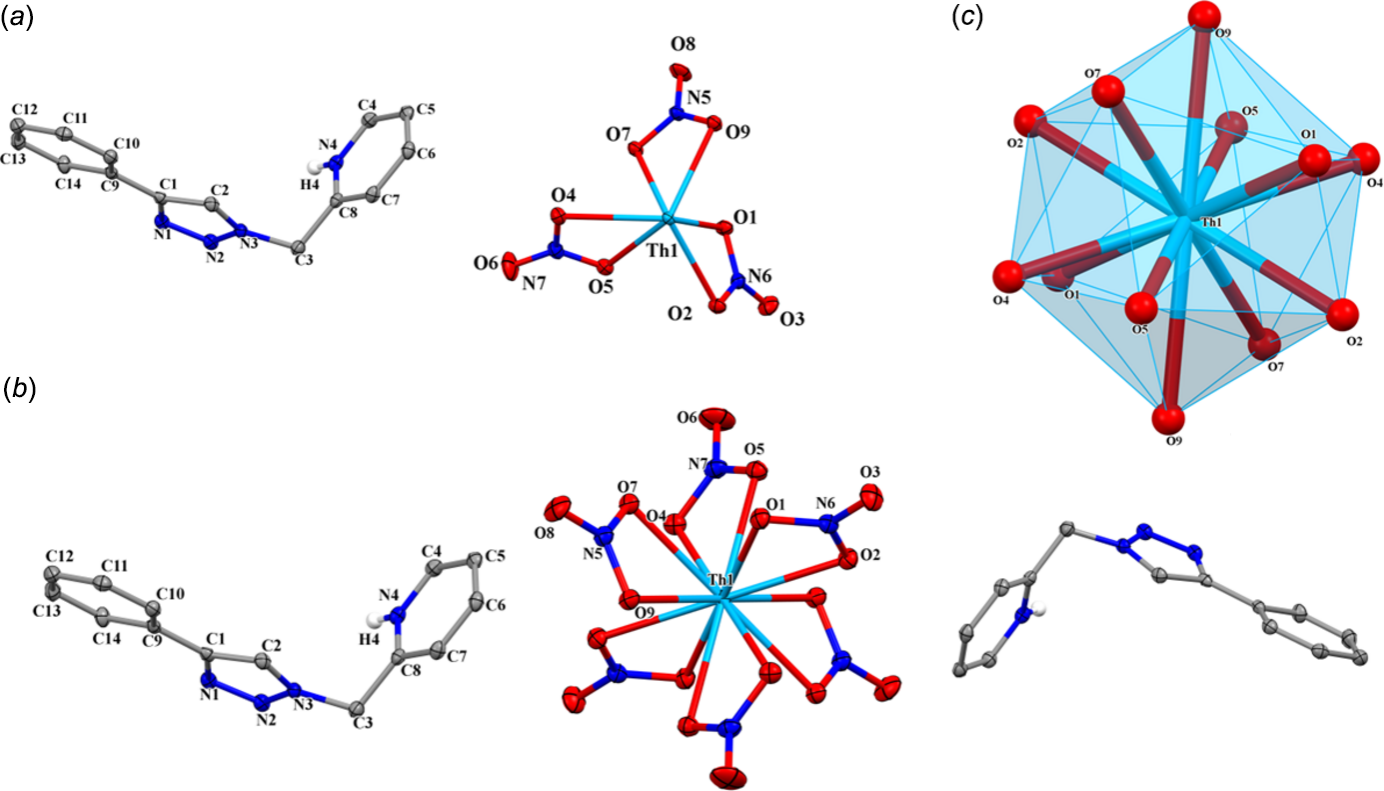
Mol­ecular structure of **1**: (*a*) Asymmetric unit of **1** showing the atom-labelling scheme, (*b*) perspective view of complex **1** and (*c*) coordination polyhedron around the Th^IV^ atom in **1**. The hydrogen atoms are omitted for clarity except for the pyridinium hydrogen. Displacement ellipsoids are drawn at the 30% probability level.

Compound **1** crystallized in the monoclinic *P*2_1_/*n* space group as a dianionic complex with two *L*H**^+^** pyridinium counter-cations, as shown in Fig. 1 ▸. The thorium atom is located on an inversion centre and is coordinated by six chelating nitrate ions to assume a distorted icosa­hedral stereochemistry (Fig. 1 ▸*c*), similar to other reported hexa­nitratothorate(IV) complexes (Abram *et al.*, 1999 ▸; Amani & Tayebee, 2013 ▸; Rammo *et al.*, 1994 ▸; Spirlet *et al.*, 1992 ▸). The Th—O bond lengths [2.5444 (13)–2.5830 (13) Å; Table 1 ▸] are similar to those reported, for example, for (H_2_ATPSC)_2_^2+^[Th(NO_3_)_6_]^2−^·4MeOH [2.553 (3)–2.580 (3) Å; HATPSC = 2-acet­ylpyridine thio­semicarbazone; Abram *et al.*, 1999 ▸], (phen_2_H^+^)_2_^2+^[Th(NO_3_)_6_]^2−^·2H_2_O [2.555 (7)–2.572 (6) Å; Amani & Tayebee, 2013 ▸], bpyH_2_^2+^[Th(NO_3_)_6_]^2−^·2H_2_O [2.567 (17)–2.599 (16) Å; Rammo *et al.*, 1994 ▸] and(NH_4_)_2_^2+^[Th(NO_3_)_6_]^2−^ [2.545 (6)–2.608 (5) Å; Spirlet *et al.*, 1992 ▸]. The pyridine nitro­gen atom of the ligand is protonated. The pyridine N—C bond lengths in **1** are N4—C8 = 1.351 (2) Å and N4—C4 = 1.340 (3) Å [1.342 (5) Å and 1.340 (5) Å in *L*; Urankar *et al.*, 2010 ▸] and the N4—H4 (pyridinium salt) bond distance is 0.862 (16) Å. In the triazole ring, the N—C distances are N1—C1 = 1.369 (2) Å and N3—C2 = 1.346 (2) Å [1.338 (5) and 1.353 (5) in *L*].

## 3. Supra­molecular features and Hirshfeld Surface Analysis

**Figure 2:**
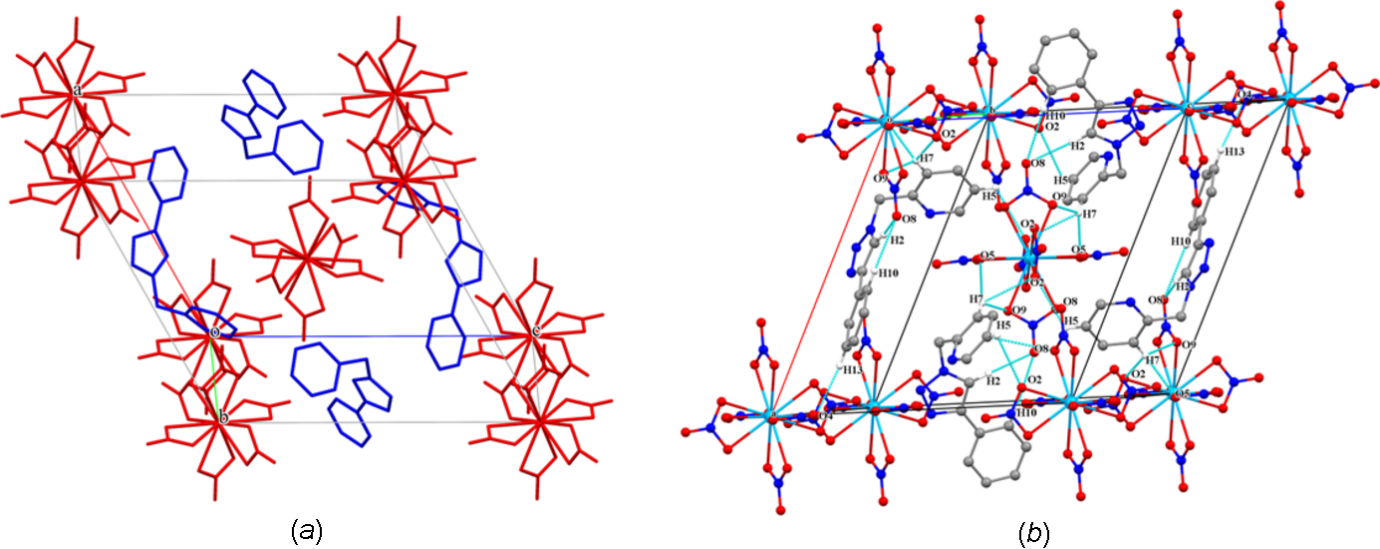
(*a*) The packing of complex **1** showing the [C_14_H_13_N_4_]^+^ cations in face-centered positions and [Th(NO_3_)_6_]^2−^ anions at the corners and in body-centered locations and (*b*) packing diagram showing inter­molecular C—H⋯O hydrogen-bonding inter­actions (blue dotted lines).

In the crystal, N—H⋯N and C—H⋯O hydrogen-bonding inter­actions are observed (Table 2 ▸). The packing also features C—H⋯π(ring) [C3—H3*A*⋯*Cg*1(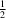 + *x*, 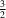 − *y*, 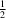 − *z*) = 3.2029 (1) Å], π(ring)–π(ring) [*Cg*1⋯*Cg*2(1 − *x*, 2 − *y*, 2 − *z*) centroid–centroid distance = 3.6130 (11) Å] and O⋯C [N6—O3⋯*Cg*3(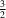 − *x*, 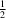 + *y*, 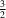 − *z*) = 3.7492 (18) Å] inter­actions where *Cg*1, *Cg2* and *Cg3* are the centroids of rings C9–C14, N1–N3/C1/C2, and N4/C4–C8, respectively. A C11⋯H3*A*(−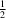 + *x*, 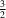 − *y*, −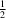 + *z*) short contact of 2.69 Å also occurs. Views of the packing of the cations and anions are displayed in Fig. 2 ▸*a* and 2*b*.

**Figure 3:**
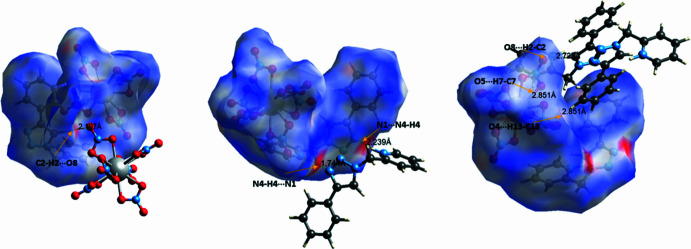
The three-dimensional Hirshfeld surface of the title compound **1**, plotted over *d*_norm_ in the range. The hydrogen bonds are shown as dashed lines.

**Figure 4:**
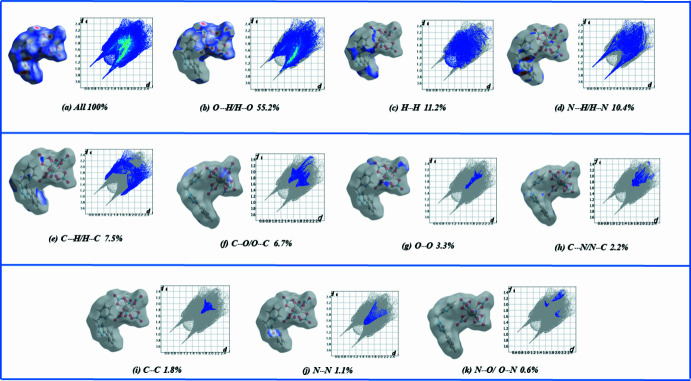
Hirshfeld surface of **1** mapped over *d*_norm_ (left images of each pair) with the corresponding two-dimensional fingerprint plots (right images of each pair). The *d*_i_ and *d*_e_ values are the closest inter­nal and external distances (in Å) from given points on the Hirshfeld surface.

In order to visualize the inter­mol­ecular inter­actions in the structure of **1**, a Hirshfeld surface (HS) analysis was carried out (Spackman & Jayatilaka, 2009 ▸) and the associated two-dimensional fingerprint plots (McKinnon *et al.*, 2007 ▸) were generated using *CrystalExplorer 17.5* (Turner *et al.*, 2017 ▸). A view of the three-dimensional Hirshfeld surface plotted over *d*_norm_ with the red, white and blue regions indicating contacts with distances shorter, equal and longer, respectively, than the van der Waals separations (Fig. 3 ▸). Inter­actions between donor and acceptor atoms are seen as red spots on the Hirshfeld surface mapped over *d*_norm_ (Fig. 3 ▸), corresponding to C2—H2⋯O8, N4—H4⋯N1, C7—H7⋯O5 and C13—H13⋯O4 hydrogen bonds. Fig. 4 ▸ shows the overall two-dimensional fingerprint plot and and those delineated into O⋯H/H⋯O (55.2%), H⋯H (11.2%), N⋯H/H⋯N (10.4%), C⋯H/H⋯C (7.5%), C⋯O/O⋯C (6.7%), O⋯O (3.3%), C⋯N/N⋯C (2.2%), C⋯C (1.8%), N⋯N (1.1%) and N⋯O/O⋯N (0.6%) inter­actions. The large number of O⋯H/H⋯O, N⋯H/H⋯H, C⋯H/H⋯C and H⋯H inter­actions suggest that hydrogen bonding and van der Waals inter­actions play a major role in the crystal packing of **1**.

## 4. Database Survey

A search of the Cambridge Structural Database (CSD, Version 5.43, last update November 2022; Groom *et al.*, 2016 ▸) for a 12-coordinate [Th(NO_3_)_6_]^2−^ moiety yielded several compounds related to the title compound, *viz.* CSD refcodes BEQVAU (Abram *et al.*, 1999 ▸), FERKOD (Cheng *et al.*, 2005 ▸), LEWNIM (Amani & Tayebee, 2013 ▸), YUWWAO (Rammo *et al.*, 1994 ▸), GOBTAS (Wang *et al.*, 1988 ▸), JOKRIN (Mishra *et al.*, 2019 ▸), LUDMIJ (Kelley *et al.*, 2020 ▸), NMPOTH (Alcock *et al.*, 1978 ▸), TEQVIX, TEQVUJ, TEQWEU, TEQWIY, TEQWUK, TEQXIZ, TEQXOF and ZIYCER01 (Jin *et al.*, 2017 ▸), UNAKAV (Goodgame *et al.*, 2003 ▸) and ZEWBOS (Aparna *et al.*, 1995 ▸). A search for lanthanide or actinide compounds with the ligand *L* did not return any hits.

## 5. Synthesis and crystallization

The ligand 2-[(4-phenyl-1*H*-1,2,3-triazol-1-yl)meth­yl]pyridine (*L*) was prepared as reported (Urankar *et al.*, 2010 ▸). Th(NO_3_)_4_·5H_2_O was purchased from a local source. Infrared spectra (4000–450 cm^−1^) of solid samples were recorded on a Bruker Alpha II instrument using the attenuated total reflection (ATR) measurement mode. The mass spectrum was recorded using a Bruker Maxis Impact LC-q-TOF Mass Spectrometer.

A solution of 2-[(4-phenyl-1*H*-1,2,3-triazol-1-yl)meth­yl]pyridine (*L*) (24 mg, 0.10 mmol) in ethanol (10 ml) was layered over a solution of Th(NO_3_)_4_·5H_2_O (57 mg, 0.10 mmol) in THF (10 ml). The reaction solution was slowly evaporated at room temperature to yield pale pink-coloured blocks of **1**. Yield = 42 mg, 78%, dec. 541 K. FT–IR (ATR cm^−1^) 2361 (*s*), 2352 (*s*), 2129 (*w*), 1632 (*s*), 1511 (*vs*, **ν_as_NO_2_**), 1472 (*s*), 1449 (*s*), 1269 (*vs*, **ν_s_NO_2_**), 1195 (*s*), 1087 (*s*), 1030 (*vs*, **νNO**), 806 (*s*), 767 (*vs*), 743 (*vs*), 708 (*s*), 694 (*s*). HRMS (*m*/*z*) calculated for C_28_H_24_N_11_O_9_Th [*M* –(2HNO_3_ + NO_3_^−^)] 890.2139; found 890.2139. Elemental analysis calculated (%) for C_28_H_26_N_14_O_18_Th (1078.63): C 31.18, H 2.43, N 18.18; found C 30.86, H 2.40, N 17.98. Owing to the poor solubility of compound **1** in organic solvents (CHCl_3_, DMSO and THF), NMR characterization could not be carried out.

The IR spectrum of **1** showed absorptions due to the nitrate ligands at 1511, 1269 and 1030 cm^−1^ (see Fig. S3 in the supporting information). The bands appearing at 1511 and 1269 cm^−1^ correspond to the asymmetric (ν_as_) and symmetric (ν_s_) NO_2_ stretching frequencies, respectively, while the band appearing at 1030 cm^−1^ is assigned to ν(NO). These values correspond with those of chelating nitrate in [Th(NO_3_)_4_·tmu] (tmu = tri­methyl­urea) at 1530, 1278 and 1023 cm^−1^ (Amani & Tayebee, 2013 ▸; Nakamoto, 2008*b* ▸) and are in contrast with those of ionic nitrates (Na and K salts), which appear at 1405–1370 cm^−1^ with ν(NO) only Raman active at 1068–1049 cm^−1^ (Nakamoto, 2008*a* ▸). The high-resolution mass spectrum of **1** showed elimination of two mol­ecules of HNO_3_ and loss of the NO_3_^−^ ion, leading to an *m*/*z* value of 820.2139 corresponding to [Th(NO_3_)_3_(C_14_H_12_N_4_)_2_]^+^, the formal ionization product of the [Th(NO_3_)_4_*L*_2_] target (see Fig. S4).

## 6. Refinement

Crystal data, data collection and refinement details are given in Table 3 ▸. C-bound hydrogen atoms were placed in calculated positions (C—H = 0.95–0.99 Å) and refined using a riding model with *U*_iso_(H) = 1.2*U*_eq_(C). The N-bound H atom H4 was refined with the distance restraint N—H = 0.89±0.02 Å.

## Supplementary Material

Crystal structure: contains datablock(s) I. DOI: 10.1107/S2056989024006352/jw2006sup1.cif

Structure factors: contains datablock(s) I. DOI: 10.1107/S2056989024006352/jw2006Isup3.hkl

Spectroscopic data and structural graphics. DOI: 10.1107/S2056989024006352/jw2006sup5.docx

CCDC reference: 2366367

Additional supporting information:  crystallographic information; 3D view; checkCIF report

## Figures and Tables

**Table 1:** Selected geometric parameters (Å, °)

Th1—O1	2.5830 (13)	N1—N2	1.314 (2)
Th1—O2	2.5621 (14)	N3—N2	1.343 (2)
Th1—O4	2.5583 (13)	N3—C2	1.346 (2)
Th1—O5	2.5547 (14)	N3—C3	1.461 (2)
Th1—O7	2.5444 (13)	N4—C4	1.340 (3)
Th1—O9	2.5827 (12)	N4—C8	1.351 (2)
N1—C1	1.369 (2)	N4—H4	0.862 (16)
			
O7—Th1—O4^i^	113.48 (4)	O9^i^—Th1—O1^i^	66.86 (4)
O7—Th1—O4	66.52 (4)	O2^i^—Th1—O9^i^	112.31 (4)
O7^i^—Th1—O9	130.23 (4)	O2^i^—Th1—O1^i^	49.70 (4)
O7—Th1—O9	49.77 (4)	O2^i^—Th1—O1	130.30 (4)
O7^i^—Th1—O1^i^	65.87 (4)	O2—Th1—O1	49.70 (4)
O7—Th1—O2^i^	69.99 (4)	O5^i^—Th1—O4	130.01 (4)
O7^i^—Th1—O5^i^	67.80 (4)	O5—Th1—O4	49.99 (4)
O4^i^—Th1—O9^i^	110.18 (4)	O5^i^—Th1—O9	66.29 (4)
O4^i^—Th1—O1^i^	112.94 (4)	O5^i^—Th1—O9^i^	113.71 (4)
O4^i^—Th1—O2^i^	113.64 (4)	O5^i^—Th1—O1^i^	69.63 (4)
O9—Th1—O9^i^	180.0	O5^i^—Th1—O2^i^	67.02 (4)

Symmetry code: (i) 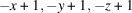.

**Table 2:** Hydrogen-bond geometry (Å, °)

*D*—H⋯*A*	*D*—H	H⋯*A*	*D*⋯*A*	*D*—H⋯*A*
N4—H4⋯N1^ii^	0.86 (3)	1.88 (3)	2.738 (3)	170 (3)
C2—H2⋯O8^iii^	0.95	2.33	3.261 (3)	168
C7—H7⋯O5^iv^	0.95	2.46	3.379 (3)	164
C13—H13⋯O4^v^	0.95	2.46	3.377 (3)	163
N4—H4⋯N2^ii^	0.86	2.65 (3)	3.432 (3)	151
C10—H10⋯O8^iii^	0.95	2.68	3.621 (3)	173
C5—H5⋯O2^i^	0.95	2.68	3.191 (2)	114
C7—H7⋯O2^iii^	0.95	2.67	3.279 (3)	123
C7—H7⋯O9^iii^	0.95	2.69	3.369 (2)	129

Symmetry codes: (i) 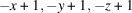; (ii) 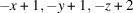; (iii) 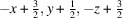; (iv) 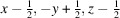; (v) 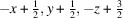.

**Table 3:** Experimental details

Crystal data	
Chemical formula	(C_14_H_13_N_4_)_2_[Th(NO_3_)_6_]
*M*_r_	1078.67
Crystal system, space group	Monoclinic, *P*2_1_/*n*
Temperature (K)	150
*a*, *b*, *c* (Å)	14.5386 (3), 9.3464 (2), 15.4213 (4)
β (°)	117.846 (1)
*V* (Å^3^)	1852.85 (7)
*Z*	2
Radiation type	Mo *K*α
μ (mm^−1^)	4.12
Crystal size (mm)	0.09 × 0.07 × 0.06

Data collection	
Diffractometer	Bruker APEXII CCD
Absorption correction	Multi-scan (*SADABS*; Krause *et al.*, 2015 ▸)
*T*_min_, *T*_max_	0.512, 0.746
No. of measured, independent and observed [*I* > 2σ(*I*)] reflections	27737, 4658, 3944
*R*_int_	0.023
(sin θ/λ)_max_ (Å^−1^)	0.673

Refinement	
*R*[*F*^2^ > 2σ(*F*^2^)], *wR*(*F*^2^), *S*	0.015, 0.035, 1.05
No. of reflections	4658
No. of parameters	281
No. of restraints	1
H-atom treatment	H atoms treated by a mixture of independent and constrained refinement
Δρ_max_, Δρ_min_ (e Å^−3^)	0.72, −0.33

Computer programs: *APEX4* and *SAINT* (Bruker, 2019 ▸), *SHELXT2014/5* (Sheldrick, 2015*a* ▸), *SHELXL2019/3* (Sheldrick, 2015*b* ▸) and *OLEX2* (Dolomanov *et al.*, 2009 ▸).

## supplementary crystallographic information

### 2-[(4-Phenyl-1H-1,2,3-triazol-1-yl)methyl]pyridin-1-ium hexakis(nitrato-κ2O,O')thorate(IV) Crystal data

**Table:**

(C_14_H_13_N_4_)_2_[Th(NO_3_)_6_]	*F*(000) = 1052
*M**_r_* = 1078.67	*D*_x_ = 1.933 Mg m^−^^3^
Monoclinic, *P*2_1_/*n*	Mo *K*α radiation, λ = 0.71073 Å
*a* = 14.5386 (3) Å	Cell parameters from 9957 reflections
*b* = 9.3464 (2) Å	θ = 3.9–28.5°
*c* = 15.4213 (4) Å	µ = 4.12 mm^−^^1^
β = 117.846 (1)°	*T* = 150 K
*V* = 1852.85 (7) Å^3^	Block, yellow
*Z* = 2	0.09 × 0.07 × 0.06 mm

### 2-[(4-Phenyl-1H-1,2,3-triazol-1-yl)methyl]pyridin-1-ium hexakis(nitrato-κ2O,O')thorate(IV) Data collection

**Table:**

Bruker APEXII CCD diffractometer	3944 reflections with *I* > 2σ(*I*)
φ and ω scans	*R*_int_ = 0.023
Absorption correction: multi-scan (SADABS; Krause *et al.*, 2015)	θ_max_ = 28.6°, θ_min_ = 2.7°
*T*_min_ = 0.512, *T*_max_ = 0.746	*h* = −19→19
27737 measured reflections	*k* = −12→12
4658 independent reflections	*l* = −20→20

### 2-[(4-Phenyl-1H-1,2,3-triazol-1-yl)methyl]pyridin-1-ium hexakis(nitrato-κ2O,O')thorate(IV) Refinement

**Table:**

Refinement on *F*^2^	Primary atom site location: dual
Least-squares matrix: full	Hydrogen site location: mixed
*R*[*F*^2^ > 2σ(*F*^2^)] = 0.015	H atoms treated by a mixture of independent and constrained refinement
*wR*(*F*^2^) = 0.035	*w* = 1/[σ^2^(*F*_o_^2^) + (0.0109*P*)^2^ + 1.5954*P*] where *P* = (*F*_o_^2^ + 2*F*_c_^2^)/3
*S* = 1.05	(Δ/σ)_max_ < 0.001
4658 reflections	Δρ_max_ = 0.72 e Å^−^^3^
281 parameters	Δρ_min_ = −0.33 e Å^−^^3^
1 restraint	

### 2-[(4-Phenyl-1H-1,2,3-triazol-1-yl)methyl]pyridin-1-ium hexakis(nitrato-κ2O,O')thorate(IV) Special details

**Table:**

Experimental. A crystal of 1 suitable for X-ray analysis was mounted on a Cryoloop with a drop of Paratone oil and placed in the cold nitrogen stream of the Kryoflex attachment of the Bruker APEX-II CCD diffractometer. The raw data was reduced with *SAINT* V8.40A (Bruker, 2019). The absorption correction was done with *SADABS2016/2* (Bruker, 2016/2). Structural solutions were obtained with SHELXT (Sheldrick, 2015a) and refined using full matrix least-squares against *F*^2^ using SHELXL (Sheldrick, 2015b), in conjunction with the Olex2 (Dolomanov *et al.*, 2009) graphical user inter­face.
Geometry. All esds (except the esd in the dihedral angle between two l.s. planes) are estimated using the full covariance matrix. The cell esds are taken into account individually in the estimation of esds in distances, angles and torsion angles; correlations between esds in cell parameters are only used when they are defined by crystal symmetry. An approximate (isotropic) treatment of cell esds is used for estimating esds involving l.s. planes.

### 2-[(4-Phenyl-1H-1,2,3-triazol-1-yl)methyl]pyridin-1-ium hexakis(nitrato-κ2O,O')thorate(IV) Fractional atomic coordinates and isotropic or equivalent isotropic displacement parameters (Å^2^)

**Table:**

	*x*	*y*	*z*	*U*_iso_*/*U*_eq_	
Th1	0.500000	0.500000	0.500000	0.01543 (3)	
O3	0.53542 (12)	0.85963 (17)	0.35224 (11)	0.0364 (3)	
O8	0.82701 (10)	0.47216 (19)	0.64969 (12)	0.0398 (4)	
O7	0.68122 (10)	0.54650 (15)	0.63986 (10)	0.0233 (3)	
O4	0.50279 (10)	0.54161 (15)	0.66518 (10)	0.0258 (3)	
O9	0.67896 (10)	0.40835 (15)	0.52678 (10)	0.0246 (3)	
O1	0.60450 (10)	0.68989 (15)	0.46103 (10)	0.0252 (3)	
O2	0.43750 (10)	0.71724 (15)	0.38557 (10)	0.0249 (3)	
O5	0.50941 (10)	0.73336 (15)	0.59005 (10)	0.0257 (3)	
N5	0.73268 (11)	0.47576 (17)	0.60683 (12)	0.0224 (3)	
N3	0.62635 (11)	0.65844 (17)	1.02726 (11)	0.0214 (3)	
N4	0.66816 (12)	0.40969 (17)	0.93013 (12)	0.0222 (3)	
N1	0.48011 (12)	0.69191 (17)	1.02356 (12)	0.0223 (3)	
N2	0.56554 (12)	0.61513 (17)	1.06624 (12)	0.0237 (3)	
C9	0.40384 (13)	0.8878 (2)	0.89961 (13)	0.0203 (4)	
N6	0.52628 (12)	0.75963 (17)	0.39768 (11)	0.0225 (3)	
N7	0.50393 (13)	0.67864 (19)	0.66342 (12)	0.0273 (4)	
C10	0.42192 (14)	0.9932 (2)	0.84549 (14)	0.0241 (4)	
H10	0.487167	0.996946	0.845381	0.029*	
O6	0.49717 (17)	0.7501 (2)	0.72531 (13)	0.0549 (5)	
C2	0.58095 (14)	0.7630 (2)	0.96064 (13)	0.0228 (4)	
H2	0.608517	0.811145	0.923638	0.027*	
C1	0.48597 (13)	0.7850 (2)	0.95776 (13)	0.0198 (3)	
C3	0.72825 (14)	0.5927 (2)	1.05898 (14)	0.0267 (4)	
H3A	0.741548	0.523286	1.112040	0.032*	
H3B	0.782565	0.667624	1.085923	0.032*	
C11	0.34551 (15)	1.0927 (2)	0.79172 (14)	0.0266 (4)	
H11	0.359140	1.165092	0.756012	0.032*	
C14	0.30661 (15)	0.8835 (2)	0.89773 (15)	0.0272 (4)	
H14	0.293076	0.812659	0.934452	0.033*	
C4	0.67031 (14)	0.3342 (2)	0.85725 (14)	0.0252 (4)	
H4A	0.620849	0.260215	0.826229	0.030*	
C8	0.73579 (13)	0.51695 (19)	0.97626 (13)	0.0206 (4)	
C5	0.74363 (14)	0.3631 (2)	0.82708 (14)	0.0248 (4)	
H5	0.746028	0.308832	0.776090	0.030*	
C6	0.81363 (15)	0.4723 (2)	0.87221 (15)	0.0258 (4)	
H6	0.864548	0.494561	0.852061	0.031*	
C7	0.80955 (14)	0.5499 (2)	0.94722 (15)	0.0238 (4)	
H7	0.857548	0.625368	0.978375	0.029*	
C12	0.24931 (16)	1.0868 (2)	0.78991 (14)	0.0290 (4)	
H12	0.196841	1.154400	0.752611	0.035*	
C13	0.23014 (16)	0.9816 (2)	0.84285 (16)	0.0317 (5)	
H13	0.164176	0.976926	0.841424	0.038*	
H4	0.6240 (17)	0.385 (3)	0.9501 (18)	0.043 (7)*	

### 2-[(4-Phenyl-1H-1,2,3-triazol-1-yl)methyl]pyridin-1-ium hexakis(nitrato-κ2O,O')thorate(IV) Atomic displacement parameters (Å^2^)

**Table:**

	*U*^11^	*U*^22^	*U*^33^	*U*^12^	*U*^13^	*U*^23^
Th1	0.01291 (4)	0.01679 (5)	0.01779 (4)	0.00037 (3)	0.00818 (3)	−0.00091 (4)
O3	0.0397 (8)	0.0323 (8)	0.0397 (8)	−0.0027 (7)	0.0206 (7)	0.0131 (7)
O8	0.0141 (6)	0.0579 (11)	0.0414 (9)	0.0031 (6)	0.0081 (6)	−0.0143 (8)
O7	0.0194 (6)	0.0259 (6)	0.0259 (7)	0.0010 (5)	0.0117 (5)	−0.0021 (6)
O4	0.0256 (7)	0.0276 (7)	0.0273 (7)	−0.0011 (5)	0.0151 (6)	−0.0020 (6)
O9	0.0204 (6)	0.0282 (7)	0.0254 (7)	0.0001 (5)	0.0109 (5)	−0.0041 (6)
O1	0.0202 (6)	0.0271 (7)	0.0280 (7)	−0.0004 (5)	0.0110 (5)	0.0012 (6)
O2	0.0198 (6)	0.0263 (7)	0.0290 (7)	0.0002 (5)	0.0116 (5)	0.0006 (6)
O5	0.0254 (7)	0.0249 (7)	0.0282 (7)	0.0002 (5)	0.0136 (6)	−0.0019 (6)
N5	0.0168 (7)	0.0258 (9)	0.0249 (8)	0.0007 (6)	0.0100 (6)	−0.0013 (6)
N3	0.0196 (7)	0.0250 (8)	0.0217 (7)	−0.0016 (6)	0.0113 (6)	−0.0010 (6)
N4	0.0189 (7)	0.0225 (8)	0.0260 (8)	−0.0010 (6)	0.0110 (6)	0.0054 (6)
N1	0.0233 (7)	0.0200 (8)	0.0274 (8)	−0.0044 (6)	0.0150 (7)	−0.0030 (6)
N2	0.0244 (8)	0.0230 (8)	0.0272 (8)	−0.0040 (6)	0.0149 (7)	−0.0021 (7)
C9	0.0209 (8)	0.0203 (9)	0.0211 (9)	−0.0029 (7)	0.0111 (7)	−0.0059 (7)
N6	0.0237 (7)	0.0220 (8)	0.0236 (8)	−0.0012 (6)	0.0126 (6)	−0.0001 (6)
N7	0.0270 (8)	0.0295 (9)	0.0271 (8)	−0.0018 (7)	0.0141 (7)	−0.0073 (7)
C10	0.0224 (8)	0.0267 (9)	0.0250 (9)	−0.0037 (8)	0.0125 (7)	−0.0042 (8)
O6	0.0897 (14)	0.0446 (10)	0.0450 (10)	−0.0058 (10)	0.0439 (10)	−0.0208 (8)
C2	0.0232 (8)	0.0257 (10)	0.0229 (9)	−0.0022 (7)	0.0136 (7)	0.0002 (7)
C1	0.0213 (8)	0.0189 (9)	0.0215 (8)	−0.0052 (7)	0.0121 (7)	−0.0055 (7)
C3	0.0199 (8)	0.0347 (11)	0.0239 (9)	0.0025 (8)	0.0090 (7)	0.0011 (8)
C11	0.0301 (10)	0.0274 (10)	0.0221 (9)	−0.0016 (8)	0.0120 (8)	−0.0006 (8)
C14	0.0260 (9)	0.0295 (10)	0.0327 (10)	0.0007 (8)	0.0193 (8)	0.0022 (8)
C4	0.0229 (9)	0.0201 (9)	0.0280 (10)	−0.0013 (7)	0.0082 (8)	0.0013 (8)
C8	0.0182 (8)	0.0207 (9)	0.0212 (8)	0.0026 (7)	0.0078 (7)	0.0045 (7)
C5	0.0272 (9)	0.0232 (9)	0.0239 (9)	0.0051 (7)	0.0119 (8)	0.0043 (8)
C6	0.0233 (9)	0.0271 (10)	0.0313 (10)	0.0033 (7)	0.0162 (8)	0.0060 (8)
C7	0.0201 (8)	0.0211 (8)	0.0309 (10)	−0.0015 (7)	0.0123 (8)	0.0018 (8)
C12	0.0291 (10)	0.0324 (11)	0.0242 (9)	0.0065 (8)	0.0112 (8)	−0.0004 (8)
C13	0.0259 (9)	0.0383 (12)	0.0363 (11)	0.0046 (8)	0.0190 (8)	0.0006 (9)

### 2-[(4-Phenyl-1H-1,2,3-triazol-1-yl)methyl]pyridin-1-ium hexakis(nitrato-κ2O,O')thorate(IV) Geometric parameters (Å, º)

**Table:**

Th1—O1	2.5830 (13)	N4—H4	0.862 (16)
Th1—O2	2.5621 (14)	C9—C10	1.393 (3)
Th1—O4^i^	2.5582 (13)	C9—C1	1.468 (3)
Th1—O4	2.5583 (13)	C9—C14	1.401 (2)
Th1—O5	2.5547 (14)	N7—O6	1.206 (2)
Th1—O7^i^	2.5444 (13)	C10—H10	0.9500
Th1—O7	2.5444 (13)	C10—C11	1.388 (3)
Th1—O9^i^	2.5827 (12)	C2—H2	0.9500
Th1—O9	2.5827 (12)	C2—C1	1.376 (2)
Th1—O1^i^	2.5830 (13)	C3—H3A	0.9900
Th1—O2^i^	2.5621 (13)	C3—H3B	0.9900
Th1—O5^i^	2.5547 (14)	C3—C8	1.507 (3)
O3—N6	1.212 (2)	C11—H11	0.9500
O8—N5	1.213 (2)	C11—C12	1.387 (3)
O7—N5	1.270 (2)	C14—H14	0.9500
O4—N7	1.281 (2)	C14—C13	1.383 (3)
O9—N5	1.277 (2)	C4—H4A	0.9500
O1—N6	1.279 (2)	C4—C5	1.374 (3)
O2—N6	1.2778 (19)	C8—C7	1.376 (2)
O5—N7	1.278 (2)	C5—H5	0.9500
N1—C1	1.369 (2)	C5—C6	1.379 (3)
N1—N2	1.314 (2)	C6—H6	0.9500
N3—N2	1.343 (2)	C6—C7	1.390 (3)
N3—C2	1.346 (2)	C7—H7	0.9500
N3—C3	1.461 (2)	C12—H12	0.9500
N4—C4	1.340 (3)	C12—C13	1.386 (3)
N4—C8	1.351 (2)	C13—H13	0.9500
			
O7^i^—Th1—O7	180.00 (6)	O8—N5—Th1	177.24 (14)
O7—Th1—O4^i^	113.48 (4)	O8—N5—O7	121.59 (16)
O7—Th1—O4	66.52 (4)	O8—N5—O9	122.56 (16)
O7^i^—Th1—O4^i^	66.52 (4)	O7—N5—Th1	57.08 (8)
O7^i^—Th1—O4	113.48 (4)	O7—N5—O9	115.85 (14)
O7^i^—Th1—O9	130.23 (4)	O9—N5—Th1	58.86 (8)
O7—Th1—O9^i^	130.23 (4)	N2—N3—C2	111.70 (15)
O7^i^—Th1—O9^i^	49.77 (4)	N2—N3—C3	120.00 (15)
O7—Th1—O9	49.77 (4)	C2—N3—C3	128.30 (16)
O7^i^—Th1—O1^i^	65.87 (4)	C4—N4—C8	122.43 (16)
O7—Th1—O1^i^	114.13 (4)	C4—N4—H4	118.1 (18)
O7^i^—Th1—O1	114.13 (4)	C8—N4—H4	119.4 (18)
O7—Th1—O1	65.87 (4)	N2—N1—C1	110.29 (15)
O7^i^—Th1—O2	69.99 (4)	N1—N2—N3	106.08 (15)
O7—Th1—O2	110.01 (4)	C10—C9—C1	120.12 (16)
O7—Th1—O2^i^	69.99 (4)	C10—C9—C14	118.62 (17)
O7^i^—Th1—O2^i^	110.01 (4)	C14—C9—C1	121.26 (17)
O7—Th1—O5	67.79 (4)	O3—N6—Th1	176.58 (13)
O7—Th1—O5^i^	112.20 (4)	O3—N6—O1	122.44 (15)
O7^i^—Th1—O5^i^	67.80 (4)	O3—N6—O2	122.05 (16)
O7^i^—Th1—O5	112.21 (4)	O1—N6—Th1	58.30 (8)
O4^i^—Th1—O4	180.0	O2—N6—Th1	57.35 (9)
O4^i^—Th1—O9^i^	110.18 (4)	O2—N6—O1	115.51 (15)
O4^i^—Th1—O9	69.82 (4)	O4—N7—Th1	57.75 (9)
O4—Th1—O9^i^	69.82 (4)	O5—N7—Th1	57.56 (9)
O4—Th1—O9	110.18 (4)	O5—N7—O4	115.19 (15)
O4^i^—Th1—O1^i^	112.94 (4)	O6—N7—Th1	174.91 (15)
O4—Th1—O1	112.94 (4)	O6—N7—O4	122.04 (18)
O4^i^—Th1—O1	67.06 (4)	O6—N7—O5	122.74 (18)
O4—Th1—O1^i^	67.06 (4)	C9—C10—H10	119.7
O4—Th1—O2	113.63 (4)	C11—C10—C9	120.61 (17)
O4^i^—Th1—O2^i^	113.64 (4)	C11—C10—H10	119.7
O4—Th1—O2^i^	66.36 (4)	N3—C2—H2	127.5
O4^i^—Th1—O2	66.37 (4)	N3—C2—C1	105.06 (16)
O9—Th1—O9^i^	180.0	C1—C2—H2	127.5
O9—Th1—O1	66.86 (4)	N1—C1—C9	123.68 (16)
O9^i^—Th1—O1	113.14 (4)	N1—C1—C2	106.87 (16)
O9^i^—Th1—O1^i^	66.86 (4)	C2—C1—C9	129.44 (17)
O9—Th1—O1^i^	113.14 (4)	N3—C3—H3A	109.2
O1^i^—Th1—O1	180.0	N3—C3—H3B	109.2
O2—Th1—O9^i^	67.69 (4)	N3—C3—C8	112.18 (15)
O2—Th1—O9	112.31 (4)	H3A—C3—H3B	107.9
O2^i^—Th1—O9^i^	112.31 (4)	C8—C3—H3A	109.2
O2^i^—Th1—O9	67.69 (4)	C8—C3—H3B	109.2
O2^i^—Th1—O1^i^	49.70 (4)	C10—C11—H11	119.9
O2^i^—Th1—O1	130.30 (4)	C12—C11—C10	120.24 (19)
O2—Th1—O1	49.70 (4)	C12—C11—H11	119.9
O2—Th1—O1^i^	130.30 (4)	C9—C14—H14	119.7
O2—Th1—O2^i^	180.0	C13—C14—C9	120.56 (19)
O5^i^—Th1—O4	130.01 (4)	C13—C14—H14	119.7
O5—Th1—O4	49.99 (4)	N4—C4—H4A	119.8
O5^i^—Th1—O4^i^	49.99 (4)	N4—C4—C5	120.40 (18)
O5—Th1—O4^i^	130.01 (4)	C5—C4—H4A	119.8
O5—Th1—O9^i^	66.29 (4)	N4—C8—C3	118.14 (16)
O5^i^—Th1—O9	66.29 (4)	N4—C8—C7	118.73 (17)
O5—Th1—O9	113.71 (4)	C7—C8—C3	123.11 (17)
O5^i^—Th1—O9^i^	113.71 (4)	C4—C5—H5	120.6
O5^i^—Th1—O1^i^	69.63 (4)	C4—C5—C6	118.76 (19)
O5—Th1—O1^i^	110.37 (4)	C6—C5—H5	120.6
O5^i^—Th1—O1	110.37 (4)	C5—C6—H6	120.1
O5—Th1—O1	69.63 (4)	C5—C6—C7	119.86 (18)
O5^i^—Th1—O2^i^	67.02 (4)	C7—C6—H6	120.1
O5—Th1—O2	67.02 (4)	C8—C7—C6	119.81 (18)
O5—Th1—O2^i^	112.98 (4)	C8—C7—H7	120.1
O5^i^—Th1—O2	112.98 (4)	C6—C7—H7	120.1
O5—Th1—O5^i^	180.0	C11—C12—H12	120.2
N5—O7—Th1	98.16 (10)	C13—C12—C11	119.64 (19)
N7—O4—Th1	97.20 (11)	C13—C12—H12	120.2
N5—O9—Th1	96.11 (9)	C14—C13—C12	120.31 (18)
N6—O1—Th1	96.79 (10)	C14—C13—H13	119.8
N6—O2—Th1	97.82 (10)	C12—C13—H13	119.8
N7—O5—Th1	97.47 (11)		
			
Th1—O7—N5—O8	177.14 (16)	C9—C14—C13—C12	−0.8 (3)
Th1—O7—N5—O9	−3.46 (16)	C10—C9—C1—N1	170.29 (17)
Th1—O4—N7—O5	−3.92 (16)	C10—C9—C1—C2	−8.1 (3)
Th1—O4—N7—O6	173.99 (18)	C10—C9—C14—C13	0.3 (3)
Th1—O9—N5—O8	−177.22 (17)	C10—C11—C12—C13	0.6 (3)
Th1—O9—N5—O7	3.39 (16)	C2—N3—N2—N1	0.5 (2)
Th1—O1—N6—O3	−176.06 (16)	C2—N3—C3—C8	−62.5 (3)
Th1—O1—N6—O2	4.18 (15)	C1—N1—N2—N3	−0.4 (2)
Th1—O2—N6—O3	176.02 (15)	C1—C9—C10—C11	−178.78 (17)
Th1—O2—N6—O1	−4.23 (16)	C1—C9—C14—C13	179.74 (19)
Th1—O5—N7—O4	3.92 (16)	C3—N3—N2—N1	−179.63 (16)
Th1—O5—N7—O6	−173.96 (18)	C3—N3—C2—C1	179.72 (18)
N3—C2—C1—N1	0.2 (2)	C3—C8—C7—C6	178.06 (17)
N3—C2—C1—C9	178.82 (18)	C11—C12—C13—C14	0.4 (3)
N3—C3—C8—N4	−59.3 (2)	C14—C9—C10—C11	0.7 (3)
N3—C3—C8—C7	122.20 (19)	C14—C9—C1—N1	−9.2 (3)
N4—C4—C5—C6	−0.9 (3)	C14—C9—C1—C2	172.4 (2)
N4—C8—C7—C6	−0.5 (3)	C4—N4—C8—C3	−178.54 (16)
N2—N3—C2—C1	−0.4 (2)	C4—N4—C8—C7	0.0 (3)
N2—N3—C3—C8	117.63 (18)	C4—C5—C6—C7	0.5 (3)
N2—N1—C1—C9	−178.62 (16)	C8—N4—C4—C5	0.7 (3)
N2—N1—C1—C2	0.1 (2)	C5—C6—C7—C8	0.2 (3)
C9—C10—C11—C12	−1.1 (3)		

Symmetry code: (i) −*x*+1, −*y*+1, −*z*+1.

### 2-[(4-Phenyl-1H-1,2,3-triazol-1-yl)methyl]pyridin-1-ium hexakis(nitrato-κ2O,O')thorate(IV) Hydrogen-bond geometry (Å, º)

*Cg*1, *Cg2* and *Cg3* are the centroids of rings C9–C14, N1–N3/C1/C2, and N4/C4–C8, respectively.

**Table:**

*D*—H···*A*	*D*—H	H···*A*	*D*···*A*	*D*—H···*A*
N4—H4···N1^ii^	0.86 (3)	1.88 (3)	2.738 (3)	170 (3)
C2—H2···O8^iii^	0.95	2.33	3.261 (3)	168
C7—H7···O5^iv^	0.95	2.46	3.379 (3)	164
C13—H13···O4^v^	0.95	2.46	3.377 (3)	163
N4—H4···N2^ii^	0.86	2.65 (3)	3.432 (3)	151
C10—H10···O8^iii^	0.95	2.68	3.621 (3)	173
C5—H5···O2^i^	0.95	2.68	3.191 (2)	114
C7—H7···O2^iii^	0.95	2.67	3.279 (3)	123
C7—H7···O9^iii^	0.95	2.69	3.369 (2)	129

Symmetry codes: (i) −*x*+1, −*y*+1, −*z*+1; (ii) −*x*+1, −*y*+1, −*z*+2; (iii) −*x*+3/2, *y*+1/2, −*z*+3/2; (iv) *x*−1/2, −*y*+1/2, *z*−1/2; (v) −*x*+1/2, *y*+1/2, −*z*+3/2.
