# Supplementary material for: Synthesis, structural studies and Hirshfeld surface analysis of 2-[(4-phenyl-1H-1,2,3-triazol-1-yl)methyl]pyridin-1-ium hexa­kis­(nitrato-κ2O,O′)thorate(IV)
Source: Acta Crystallogr E Crystallogr Commun. 2024 Jul 5;80(Pt 8):820–5. doi: 10.1107/S2056989024006352 (PMC11299750; doi:10.1107/S2056989024006352)
Supplement: Supplementary file 3 [file e-80-00820-sup5.docx]

**Electronic Supplementary Data**

**Synthesis, structural studies** **and Hirshfeld surface analysis of 2-((4-phenyl-1H-1,2,3-triazol-1-yl)methyl)pyridin-1-ium hexakis(nitrato-*O*,*O'*)- thorate**


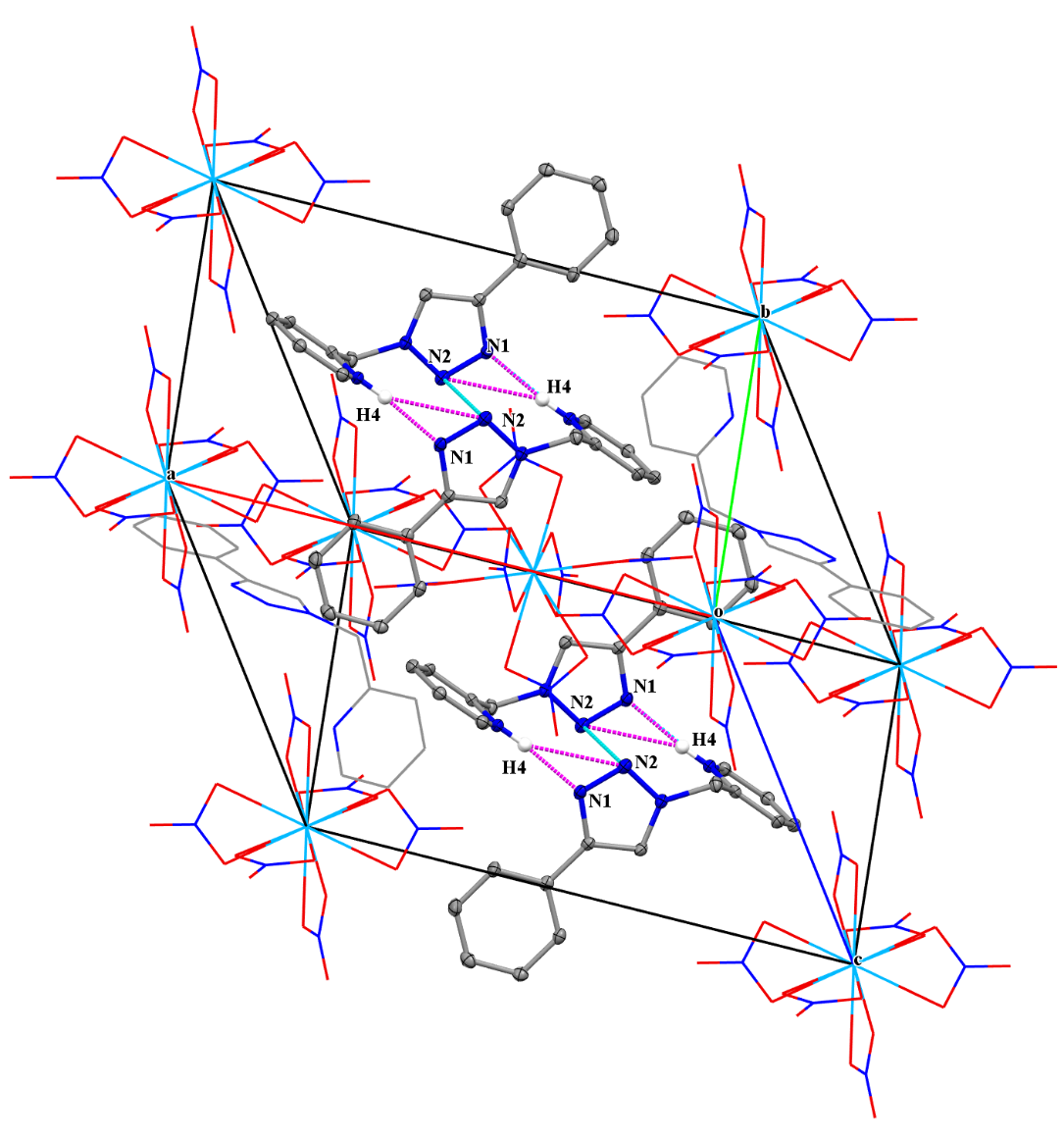


**Figure S1**

Packing diagrams showing the two-dimensional arrangement of **1** showing intermolecular H-bonding N‧‧‧H-N (pink dotted lines) along *a*- and *c*- axes direction and N···N contacts along *a*- axis direction.


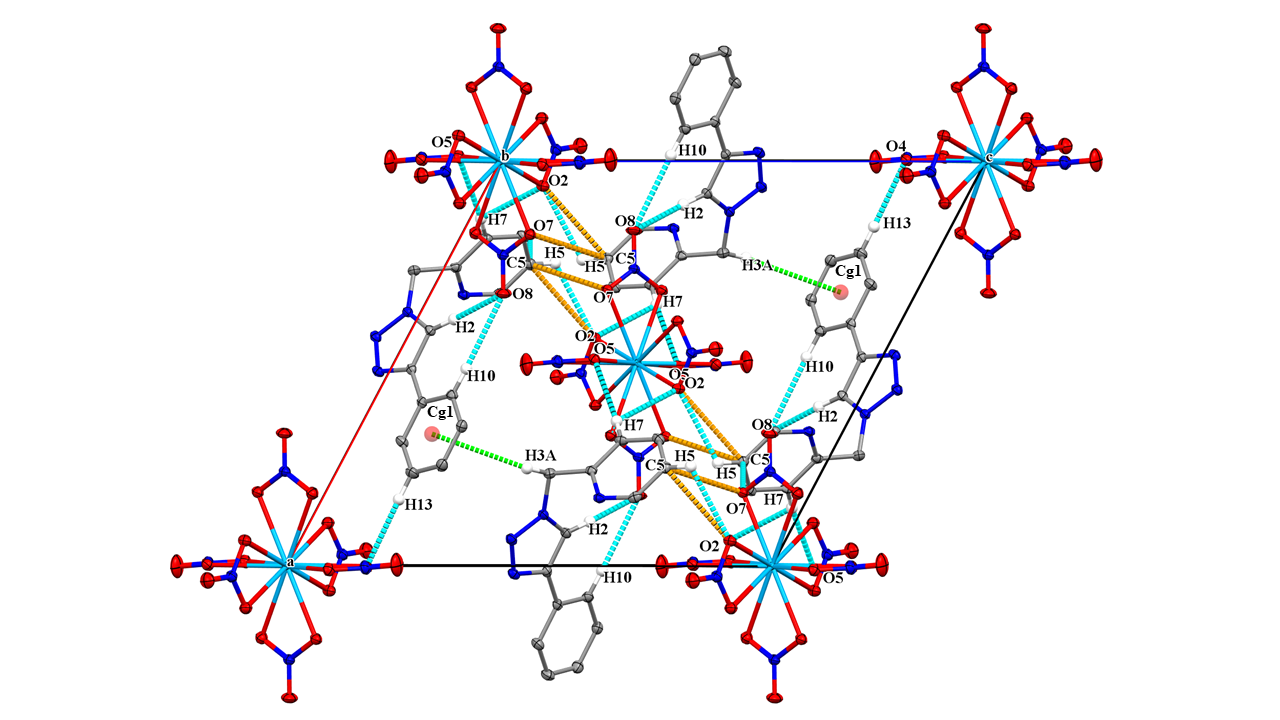


**Figure S2**

Packing diagrams showing the two-dimensional arrangement of **1** showing π···H interactions (green dotted lines) along *b*- and *c*-axes direction, O‧‧‧H interactions (blue dotted lines) along *a*- and *b*-axes direction and O‧‧‧C contacts (yellow dotted lines) along *b*- axis direction.


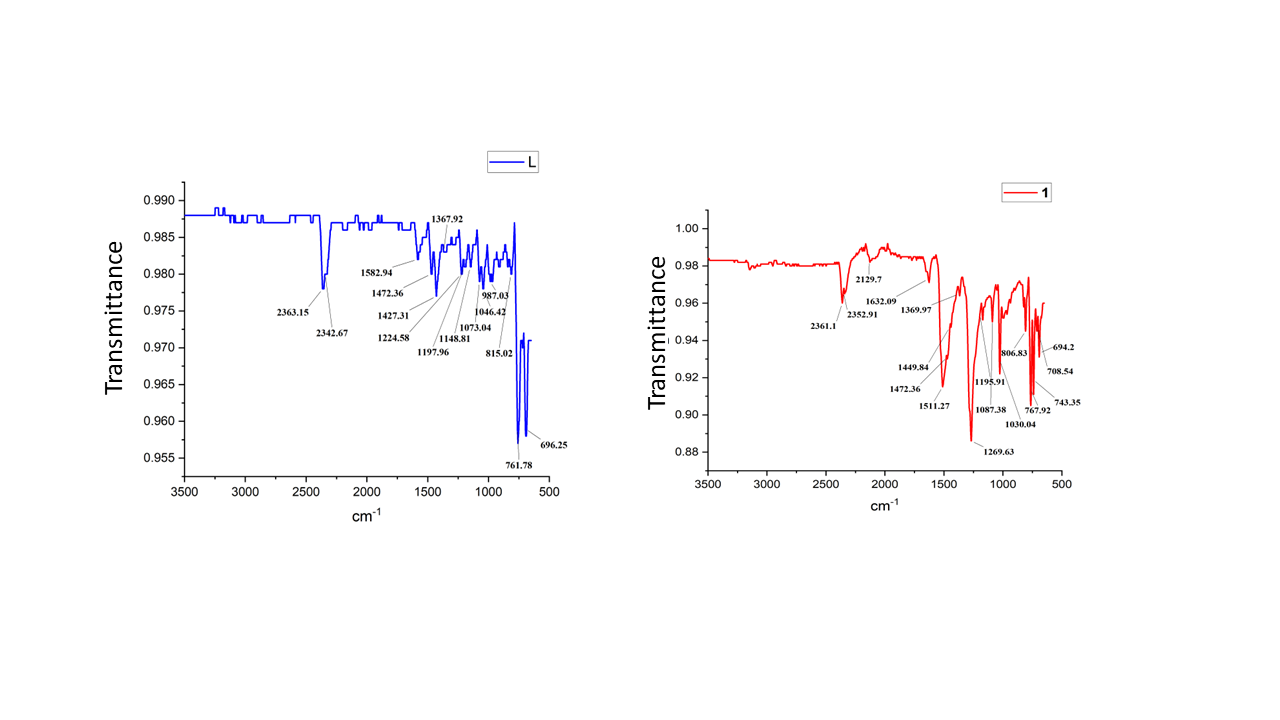


**Figure S3**

IR spectra of compounds **L** and **1**.


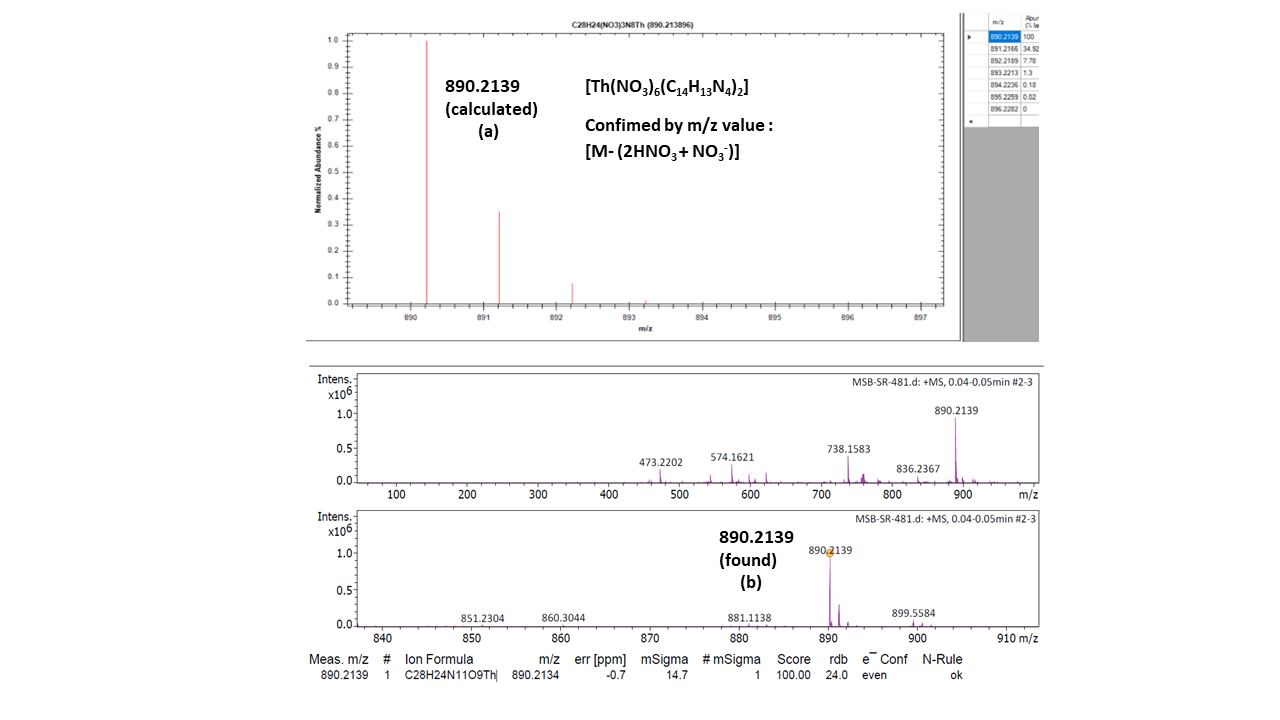


**Figure S4** HRMS spectra of **1** a) calculated and b) found.

**Table S1** Comparison of **H···*A*** values for Table 2 and Fig 3

| ***D*—H···*A*** | **H···*A* (Table 2)** | **H···*A* (Fig 3)** |
| --- | --- | --- |
| C2—H2···O8 | 2.33 | 2.20 |
| N4—H4···N1 | 1.88 | 1.74 |
| C7—H7···O5 | 2.46 | 2.85 |
| C13—H13···O4 | 2.46 | 2.85 |
